# A two-stage maintenance trial of cetuximab-based treatment in RAS and BRAF wild-type unresectable metastatic colorectal cancer: a retrospective real-world study

**DOI:** 10.3389/fonc.2024.1425203

**Published:** 2024-07-23

**Authors:** Tao Jiang, Hao Chen, Xinli Wang, Fangyu Lin, Han Wang, Jialin Liu, Xiaoyan Lin

**Affiliations:** ^1^ Department of Medical Oncology, Fujian Medical University Union Hospital, Fuzhou, China; ^2^ Fujian Provincial Key Laboratory of Translational Cancer Medicine, Fuzhou, China

**Keywords:** FOLFIRI, RAS and BRAF, colorectal cancer, real-world study, cetuximab

## Abstract

**Background:**

To investigate the effectiveness and safety of maintenance regimens based on cetuximab, we conducted a real-world, single-arm, retrospective study at a single center.

**Methods:**

In Fujian Medical University Union Hospital, patients with unresectable metastatic colorectal cancer (mCRC) who received cetuximab-based maintenance therapy between December 2020 and December 2021 were included. All patients had RAS and BRAF wild-type. The maintenance regimen consisted of 6–12 cycles of cetuximab plus irinotecan (Phase 1) and cetuximab (Phase 2). Patients could receive reintroduction therapy in case of disease progression during Phase 2. Progression-free survival (PFS), overall survival (OS), and safety data were collected.

**Results:**

According to the inclusion and exclusion criteria of the study, a total of 108 subjects who received maintenance therapy were included— 51 experienced disease progression during Phase 1, with PFS (1) of 7.3 months. Among the 52 patients who entered Phase 2, 17 were still in this phase at the end of follow-up, with PFS (2) of 10.1 months. In Phase 2, 35 patients experienced disease progression, of whom 24 received reintroduction therapy, with PFS (3) of 6.7 months. The overall PFS (total) during the maintenance period was 11.9 months, and the OS was 39.2 months. Grade III or higher adverse events were 4.6% during Phase 1 and 0% during Phase 2.

**Conclusion:**

Innovative cetuximab-based maintenance therapy showed a trend toward improving the prognosis of mCRC patients with RAS and BRAF wild-type, while the toxic side effects of maintenance therapy were manageable.

**Clinical trial registration:**

https://www.chictr.org.cn, identifier ChiCTR2000040940.

## 1 Background

Colorectal cancer (CRC) is a prevalent malignancy of the digestive tract (1), typically diagnosed at an advanced stage due to the absence of obvious early symptoms. Approximately 20% of colorectal cancer patients lose the opportunity for radical surgery (2, 3). Currently, for metastatic CRC (mCRC) patients with non-microsatellite instability (non-MSI), chemotherapy plus targeted therapy is the standard treatment regimen. For patients with *RAS* and *BRAF* wild-type, especially those with primary tumors located in the left colon or rectum, cetuximab has demonstrated superiority over bevacizumab in terms of early tumor shrinkage (ETS), depth of tumor response (DPR), disease control rate (DCR), and overall survival (OS) (4, 5). Clinical studies have recommended continuing first-line therapy with cetuximab plus chemotherapy until disease progression or intolerable toxicity (6–8).

However, continuous chemotherapy can lead to drug toxicity accumulation and increased treatment resistance (9). Conversely, discontinuing treatment increases the likelihood of disease progression (10, 11). To avoid these adverse outcomes, maintenance therapy is introduced to maintain a balance between drug efficacy and toxicity. Although 5-fluorouracil(5-FU) or capecitabine with or without bevacizumab is the predominant maintenance regimen (12, 13), the optimal model for cetuximab-based maintenance remains to be explored. Therefore, the objective of this study was to evaluate the effectiveness and safety of cetuximab-based step-down maintenance treatment protocols within a retrospective, real-world analysis.

A pilot study (14) (Registration Number ChiCTR1900026360) revealed that cetuximab-based maintenance therapy is well-tolerated and was associated with prolonged failure-free survival (FFS) of mCRC patients compared with observation without further interventions. Based on these findings, the data of mCRC patients who received first-line therapy with cetuximab plus FOLFIRI, followed by cetuximab-based step-down maintenance therapy, were collected to analyze the efficacy and safety of cetuximab-based maintenance therapy in clinical practice.

## 2 Materials and methods

### 2.1 Study design

This retrospective real-world study was approved by the Ethics Review Committee of Fujian Medical University Union Hospital (Fuzhou City, Fujian Province, China; Number 2020KY0144), and the clinical study registration number is ChiCTR2000040940.

Patients’ data were collected according to inclusion and exclusion criteria. Inclusion criteria: (1) Patients with unresectable mCRC treated at Fujian Medical University Union Hospital from January 1, 2016, to January 1, 2020. (2) RAS and BRAF genes were wild-type by histological examination (detection sites included exons 2–4 of *KRAS* and *NRAS* genes and *V600E* of the BRAF gene). (3) Received first-line induction therapy with cetuximab + FOLFIRI [cetuximab: 500 mg/m^2^ (d1) + irinotecan: 180 mg/m^2^ (d1) + calcium folinate: 400 mg/m^2^ (d1) + 5-FU: 2.4 g/m^2^ 46 h, every two weeks] for 9–12 cycles, and the therapeutic response was evaluated by imaging and laboratory examinations to reach stable disease (SD), partial response (PR), or complete response (CR). (4) Received the cetuximab-based maintenance therapy after the first-line induction treatment. Exclusion criteria: 1. Complete clinical data could not be collected. 2. Disease progression during the first-line induction therapy.

**Figure 1:**
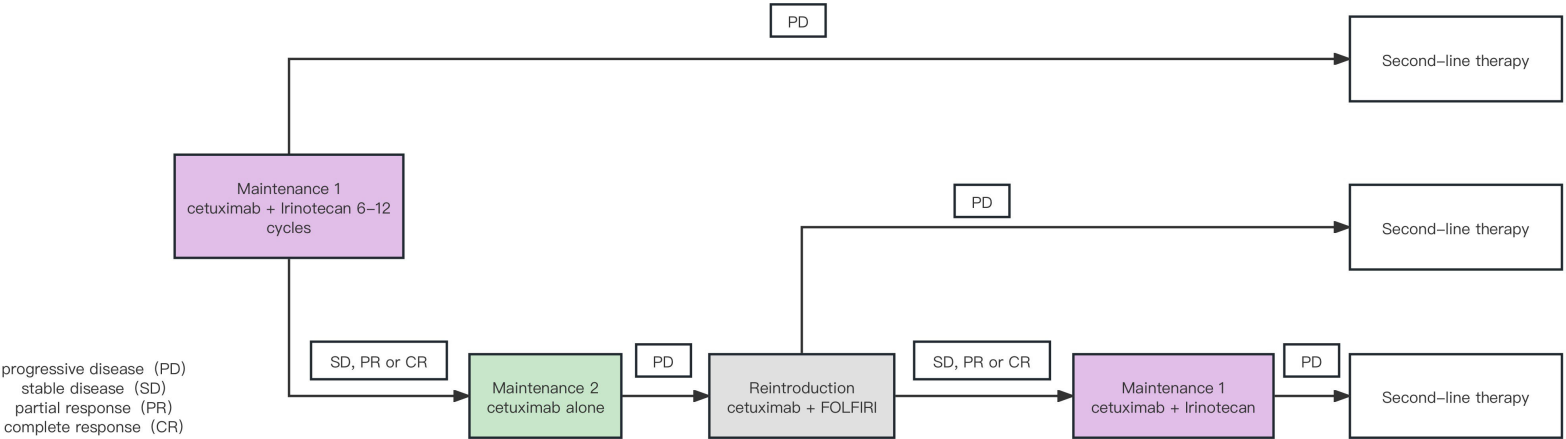
Retrospective real-world study design for maintenance therapy.

The maintenance regimen was conducted in two phases: 1) Maintenance Phase 1: This phase involved the combination therapy with cetuximab [500 mg/m^2^ (d1, every two weeks)] and irinotecan [180 mg/m^2^ (d1, every two weeks)] for 6–12 cycles. 2) Maintenance Phase 2: Patients who responded to the combination therapy (SD, PR, or CR) in Maintenance Phase 1 entered this phase, which involved treatment with cetuximab alone [500 mg/m^2^ (d1, every two weeks)]. If the Maintenance Phase 2 efficacy evaluation shows Progressive Disease (PD), patients could receive first-line reintroduction therapy with cetuximab plus FOLFIRI (9–12 cycles) and step-down maintenance therapy until either reintroduction failure or disease progression during Maintenance Phase 1 and then enter second-line therapy, which did not limit the therapeutic regimen. The design of the above maintenance protocol is detailed in **Figure 1**.

According to Response Evaluation Criteria in Solid Tumors version 1.1, the patient’s responses to treatment were evaluated every 3–4 cycles through systematic assessment. Endpoints were evaluated for all treatment periods, including Maintenance Phase 1, Maintenance Phase 2, Reintroduction therapy, and Maintenance treatment. Adverse events were graded according to the National Cancer Institute Common Toxicity Criteria version 5.0. Patients were followed up until June 30, 2022, or death using medical records, outpatient clinics, and telephone calls. The study’s primary endpoints were Progression-free survival (PFS), and secondary endpoints included OS, objective response rate (ORR), DCR, and treatment safety.

Statistical data were analyzed using SPSS 25.0. The baseline characteristics of patients and disease risk factors were summarized using descriptive statistics and analyzed using the t-test. In addition, categorical data were analyzed using the χ^2^ test. The Kaplan-Meier method was adopted to measure the median survival time. Hazard ratios (HRs) were determined using Cox regression, and P-values were determined using the log-rank test. P<0.05 was considered statistically significant.

### 2.2 Meta-analysis

A systematic search was performed using PubMed, EBSCO, Cochrane Library, CNKI, VIP Chinese Journal of Science and Technology, and the Wanfang database platform, and all titles and abstracts involved were evaluated and screened to determine that they met the inclusion requirements. Inclusion criteria included: (1) Prospective cohort study and randomized controlled trial. (2) The study population included patients with histologically or cytologically confirmed RAS and BRAF wild-type mCRC, excluding appendiceal or anal canal cancer. (3) Patients who have received cetuximab-based induction or maintenance therapy after the diagnosis of mCRC are expected to be followed for more than three months. (4) When multiple articles from the same study are identified, these two articles can be included together if the reported results are different. (5) When multiple articles conducted in the same study population reported the same results, the article with the longest follow-up time was selected. (6) Excluding published abstracts. The search covered the period from database inception to October 2022. For studies that met the inclusion criteria, Revman 5.3 was used for meta-analysis of the data.

## 3 Results

### 3.1 Meta-analysis

A meta-analysis was conducted to investigate the current research status of maintenance regimens. The analysis included three RCTs with 644 patients (6, 7, 15). The results, presented in **Supplementary Figure 1**, showed that the risk of disease progression was significantly lower in the group with continuous anti-EGFR monoclonal antibody (mAb) therapy than in the group without continuous anti-EGFR mAb therapy (P<0.00001).

### 3.2 Real-world data analysis

**Figure 2:**
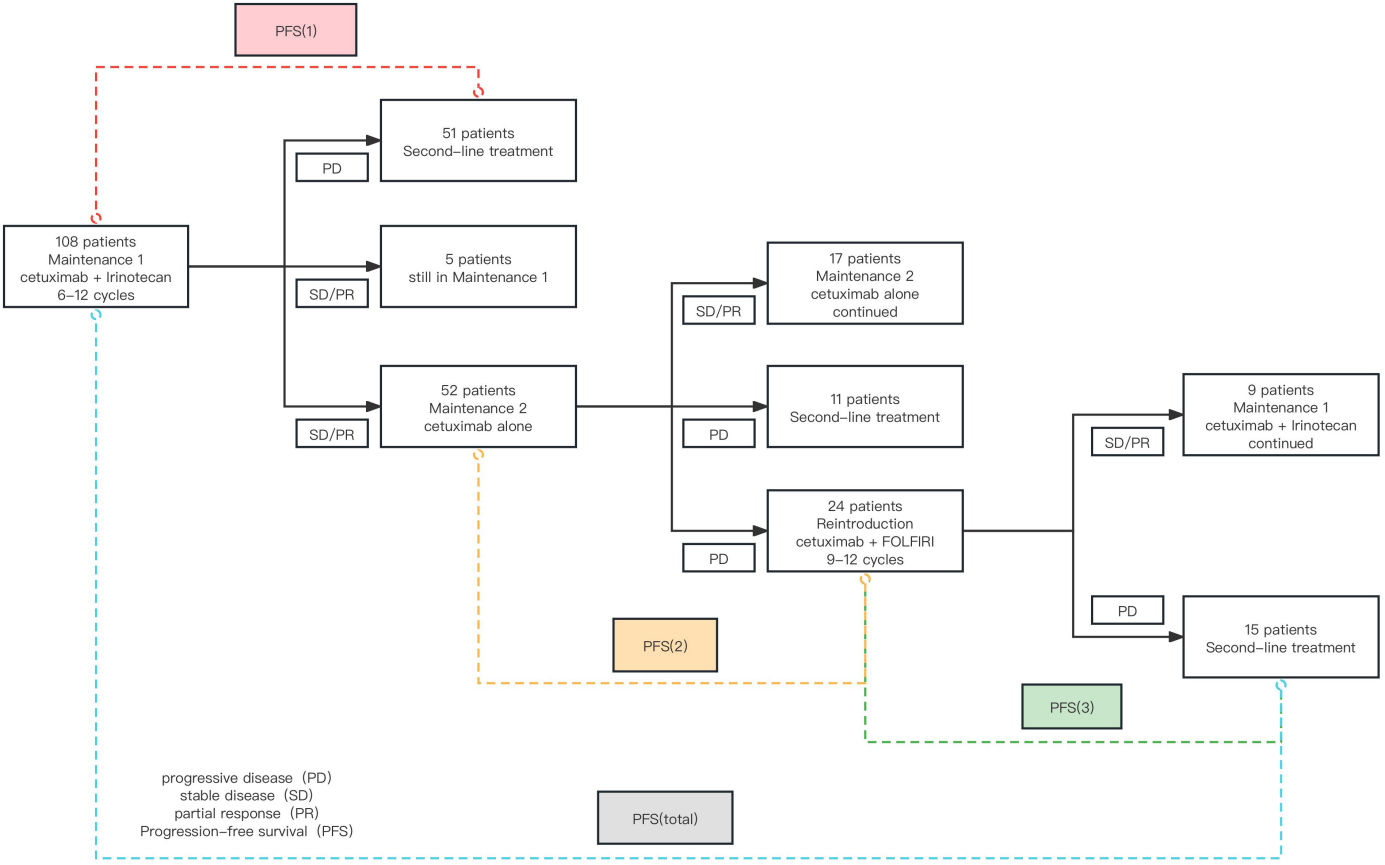
Flowchart Illustrating the Step-Down Maintenance Therapy Protocol.

Following the study protocol, out of 127 patients with metastatic colorectal cancer (mCRC) who underwent first-line induction therapy with cetuximab and FOLFIRI, we identified 108 individuals who fulfilled the specified inclusion and exclusion criteria. The remaining 19 patients exhibited disease progression while on induction therapy. Subsequently, 108 patients who met the criteria advanced to the step-down maintenance phase. During Maintenance Phase 1, 51 patients experienced disease progression, leading to the initiation of second-line therapy. At the same time, five patients continued in Maintenance Phase 1. According to the efficacy evaluation criteria, the remaining 52 patients completed 6 to 12 cycles, achieving either SD or PR. Subsequently, these 52 patients proceeded to Maintenance Phase 2. After the follow-up period, 17 patients were still ongoing in Maintenance Phase 2. Of the remaining 35 patients who experienced PD, 24 underwent a reintroduction of the cetuximab plus FOLFIRI regimen. The patients’ conditions during step-down maintenance therapy are depicted in **Figure 2**.

All patients who underwent maintenance treatment were followed for survival status up to June 30, 2022, demonstrating a 100% rate of follow-up for PFS. However, the OS follow-up rate was 96.3%, as four patients did not have available follow-up OS records. Among the 108 patients, 66 were male, and 42 were female, aged 27–79. There were 12 cases of right-side colon cancer (ileocecal junction to splenic flexure) and 96 cases of left-side colorectal and rectal cancer (from splenic flexure to sigmoid colon and rectum). The Eastern Cooperative Oncology Group (ECOG) score ranged from 0 to 2. Primary tumor resection was performed on 30 of the 108 patients. Forty-seven patients had liver metastasis only, 13 patients had lung metastasis only, 13 patients had both liver and lung metastasis, and the rest had metastasis in other organs. During maintenance therapy, 42 patients received local treatment of metastatic lesions, including radiofrequency ablation for lung metastasis in 3 patients (surgical resection in 1 patient), liver metastasis in 25 patients, simultaneous liver and lung metastasis in 5 patients, and metastasis at other sites in 9 patients (surgical resection in 1 patient). The patients’ baseline data are shown in **Table 1**.

**Table 1:** Patient baseline information form.

	Primary analysis population
Variable	maintenance treatment (n=108)
Male sex (%)	61.1
Age(years)	
Median	57
Range	27–79
ECOG performance status (%)	
0	55.5
1	41.7
2	2.8
Primary tumor (%)	
Left colon	88.9
Right colon	11.1
Resection	27.8
No resection	72.2
Metastases (%)	
Lung	12.0
Liver	43.5
Both lung and liver	12.0
Multiple metastases	32.5
Treatment of metastases	38.9
Without treatment	61.1

The efficacy in each treatment stage is shown in **Supplementary Table 1**. 108 patients received step-down Maintenance Phase 1 treatment, with a DCR of 48.1% and an ORR of 11.1%. Fifty-two patients who entered step-down Maintenance Phase 2 achieved SD or PR and showed a DCR of 32.7% and an ORR of 11.5%. Twenty-four patients who received reintroduction therapy due to PD during Maintenance Phase 2 showed a DCR of 37.5% and an ORR of 12.5%. No patient’s efficacy evaluation achieved CR throughout the maintenance treatment phase.

**Figure 3:**
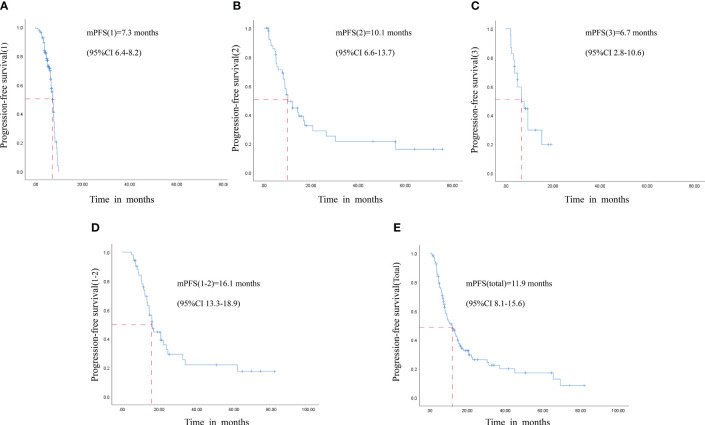
Plot of patient survival on step-down maintenance therapy. **(A)** Maintenance Phase 1 mPFS(1). **(B)** Maintenance Phase 2 mPFS(2). **(C)** Reintroduction therapy mPFS(3). **(D)** Maintenance Phases 1 and 2 mPFS(1–2). **(E)** mPFS (total) for all maintenance patients. mPFS: median progression-free survival. CI, Confidence interval.

The median PFS (1) for patients in Maintenance Phase 1 was 7.3 months, with a 95% confidence interval (CI) of 6.4 to 8.2, as shown in **Figure 3A**. Patients in Maintenance Phase 2 exhibited a median PFS (2) of 10.1 months (95%CI: 6.6–13.7), depicted in **Figure 3B**. Furthermore, those who underwent reintroduction of the cetuximab plus FOLFIRI regimen had a median PFS (3) of 6.7 months (95% CI 2.8–10.6), presented in **Figure 3C**. A pooled analysis of follow-up data from both maintenance phases revealed that the median PFS (1–2) for the 52 patients who completed therapy in Maintenance Phases 1 and 2 was 16.1 months (95%CI: 13.3–18.9), as illustrated in **Figure 3D**. Considering all 108 patients who underwent maintenance therapy, the overall median PFS (total) was 11.9 months, within a 95% CI of 8.1 to 15.6, as shown in **Figure 3E**. Adverse reactions during Maintenance Phases 1 and 2 are shown in **Supplementary Figures 2**, **3**. Grade III or higher adverse events were 4.6% during Phase 1 and 0% during Phase 2. Analyzing the survival data from all enrolled patients, the median OS was determined to be 39.2 months (95%CI: 36.0–56.1), as detailed in **Supplementary Figure 4**. The respective 2-year and 3-year survival rates were 47.1% and 23.1%.

**Figure 4:**
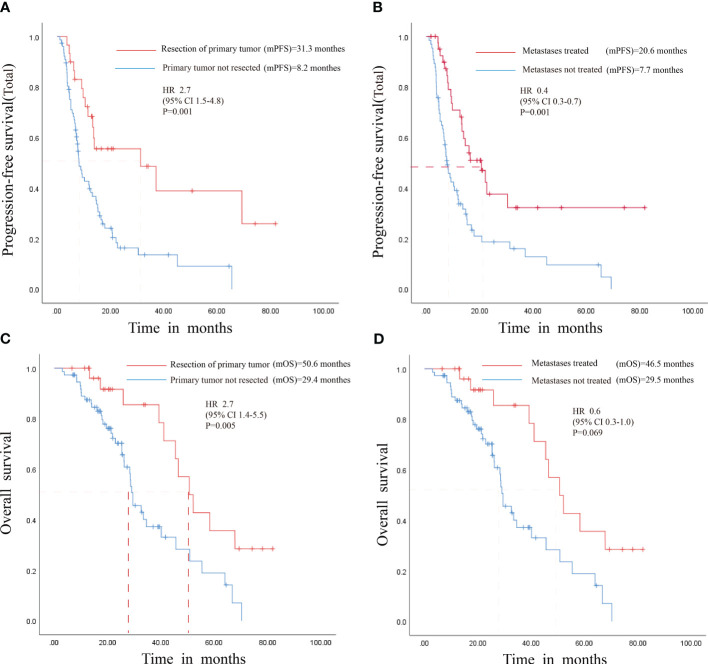
Survival Analysis of Patients with Resected Primary Tumors and Locally Treated Metastases. **(A)** PFS for patients with primary tumor resection. **(B)** PFS for patients with local treatment of metastases. **(C)** OS for patients with primary tumor resection **(D)** OS for patients with local treatment of metastases. HR, Hazard Ratio; mPFS, median progression-free survival; mOS, median overall survival; CI, Confidence interval.

Correlation analysis was performed to investigate the associations between the patients’ clinical data and their PFS and OS outcomes. The analysis revealed significant prolongation in PFS for patients with metachronous metastases following primary tumor resection and for those undergoing local treatment of metastases while on maintenance therapy. Concurrently, OS was significantly extended in patients with primary tumor resection. However, no discernible OS benefit was observed in patients receiving local metastase therapy (**Figure 4**) (**Supplementary Tables 2**, **3**).

## 4 Discussion

Balancing the risk of disease progression with the impact of the discontinuation of treatment on quality of life and treatment compliance is challenging for mCRC patients (12, 13). Maintenance therapy with effective and less toxic drugs is one strategy to address this dilemma. Nevertheless, the optimal maintenance regimen after induction therapy remains a matter of debate.

Cetuximab is an IgG1 type mAb that competes with the EGFR transduction pathways to exert antitumor effects in mCRC. Large-scale RCTs have demonstrated that cetuximab plus chemotherapy significantly benefit patients with *RAS* gene-wild-type mCRC, especially on the left side, regarding ETS, DPR, DCR, and OS (16, 17). Given that previous clinical studies and guidelines have recommended that cetuximab be continuously received by patients until disease progression, it is essential to explore its value as maintenance therapy after first-line chemotherapy. In the European Society for Medical Oncology (ESMO) guidelines (2022), 5-FU in combination with anti-EGFR mAb is recommended as a regimen for maintenance therapy (II, B) (18). However, no phase III data supports maintenance treatment with anti-EGFR mAbs.

The author’s team has conducted a pilot retrospective study investigating the efficacy of maintenance therapy in mCRC (Registration No. ChiCTR1900026360) (14). The results revealed that cetuximab-based maintenance therapy is well-tolerated and was associated with prolonged failure-free survival (FFS) of mCRC patients compared with observation (12.7 vs. 3.0 months; HR=0.202; P<0.001). Patients may also benefit from a reintroduction regimen after disease progression in maintenance therapy (19). A retrospective real-world study on cetuximab-based maintenance therapy was conducted on this basis.

The meta-analysis revealed that mCRC patients who received cetuximab-based continuation therapy had a lower risk of disease progression than those who did not (**Supplementary Figure 1**). Furthermore, additional scholars have reported analogous findings in meta-analyses, indicating a trend toward enhanced benefits from continuing cetuximab-based maintenance therapy following induction chemotherapy combined with cetuximab (20). These results suggested that further research is needed to evaluate the effectiveness and safety of cetuximab as maintenance therapy.

In devising our maintenance treatment strategy, we opted for a combination of irinotecan and cetuximab, influenced by several key considerations: (1) Given that the patient’s induction phase incorporated cetuximab with the FOLFIRI regimen, selecting maintenance agents from this proven practical treatment approach was deemed more suitable. (2) Utilizing a 5-FU chemotherapy pump for maintenance did not offer a reduction in patient hospital stays. (3) The administration schedules of capecitabine and cetuximab were not completely aligned, and capecitabine may cause a certain degree of hand-foot syndrome. Furthermore, although capecitabine is also a drug in the 5-FU class, it is still considered a switch to a different medication. (4) The cumulative neurotoxicity of oxaliplatin results in relatively poor patient tolerance. Irinotecan has been successfully applied in clinical practice among the Chinese population. Therefore, we have chosen a maintenance treatment plan that pairs irinotecan with cetuximab.

Statistical analysis indicates that cetuximab-based step-down maintenance therapy is promising in improving patients’ prognoses with RAS and BRAF wild-type mCRC. Concurrently, the toxic side effects associated with this maintenance therapy are deemed manageable. In Maintenance Phase 1, following the discontinuation of 5-FU and the continued administration of cetuximab and irinotecan, the PFS (1) was observed to be 7.3 months. The patients tolerated this therapeutic approach well. Patients who entered Maintenance Phase 2 had a PFS (2) of 10.1 months, leading to reduced incidence rates of delayed diarrhea and cholinergic syndrome due to discontinuing irinotecan. Reintroducing the initial first-line induction regimen for patients who progressed on Maintenance Phase 2 resulted in a PFS (3) of 6.7 months. Reintroduction was a suitable option for patients who experienced disease progression during maintenance therapy with cetuximab alone. Prognostic analysis of all patients who entered the maintenance regimen showed that the PFS (total) was 11.9 months and the OS was 39.2 months, 2-year OS of 47.1%, and 3-year OS of 23.1%, exhibiting good DCR and ORR and better benefits than other published maintenance regimens. In addition, correlation analysis suggests that maintenance therapy improved the PFS in patients with mCRC, irrespective of whether they have undergone primary tumor resection or received local treatment for metastases. Furthermore, while maintenance therapy appears to influence OS in patients with their primary tumor resected positively, those treated locally for metastases do not demonstrate a significant trend toward enhanced OS benefits. This may be related to insufficient sample size and warrants future analysis of large clinical trials.

The survival follow-up data also showed that maintenance therapy prolonged OS, particularly in patients entering Maintenance Phase 2. Compared with non-contemporaneous Fire-3 and CALGB/SWOG80405 studies (9, 17), we found a striking feature: OS was prolonged in patients included in maintenance therapy. This indicates that patients on maintenance therapy exhibit a favorable trend in disease progression and survival outcomes, with the treatment being well tolerated.

At the same time, these patients also showed a trend towards higher survival rates at 2 and 3 years. This finding highlights the positive effect of maintenance therapy in improving patient survival time and survival. However, it is essential to note that this study is a retrospective real-world single-arm study, and further head-to-head phase II or III clinical trials are still needed for further validation in the future.

Several studies have investigated the effectiveness of cetuximab-based maintenance therapy. The phase II MACBETH study (21) compared cetuximab and bevacizumab-based maintenance therapy after induction therapy but did not meet the primary endpoint. The COIN-B study (22) compared cetuximab-based maintenance therapy with observation after induction therapy and showed that the cetuximab-based maintenance group had a longer FFS (14.3 vs. 12.2 months). Subsequently, the results of the PRODIGE-28 study showed that cetuximab maintenance therapy tended to prolong the PFS and had a favorable safety profile compared with observation. The MACRO-2 TTD study (23) revealed that cetuximab-based maintenance therapy equals the original continuous treatment regimen, but further verification is still needed. Chinese scholars have also carried out several relevant studies on cetuximab-based maintenance therapy, and a similar effectiveness of cetuximab combined with single-agent maintenance chemotherapy was observed (24, 25). In addition, the phase III CLASSIC study was performed to explore the efficacy and safety of cetuximab in combination with capecitabine versus cetuximab alone as maintenance therapy. Cetuximab has demonstrated initial effectiveness in the maintenance treatment, but the beneficiary population is mainly RAS and BRAF wild-type patients. Since patients may experience RAS gene-phenotype conversion during cetuximab-based maintenance therapy (26), dynamic monitoring of RAS phenotype through circulating tumor DNA (ctDNA) during maintenance therapy is worthy of further clinical exploration.

The optimal maintenance treatment model for mCRC is still being explored. The OPTIMOX-1 trial (27) showed that discontinuation of oxaliplatin could be considered after six cycles of FOLFOX induction. The subsequent OPTIMOX-2 study showed that 5-FU-based maintenance therapy significantly prolonged PFS and OS compared with intermittent therapy (10). Waddell et al. (28) found that sequential capecitabine-based maintenance therapy after short-course XELOX chemotherapy can be an effective, low-toxic, and convenient option. Xu et al. (29) found that capecitabine maintenance significantly prolonged PFS compared with observation, but the difference was insignificant in OS. In addition, the FOCUS4-N study (30) reported that PFS was significantly prolonged in the capecitabine-based maintenance group compared with that in the observation group. However, the median OS showed no evident difference. Although the NO. 16966 trial (31) first supported bevacizumab-based maintenance therapy, subsequent studies like SAKK41/06 and PRODIGE-9 (32, 33) did not conform to its superiority over discontinuation observation. The MACROTTD study (34) compared the efficacy of bevacizumab-based maintenance therapy with bevacizumab + XELOX continuation regimen, which did not meet the pre-specified non-inferiority endpoint. The AIO0207 research (35) confirmed the advantage of bevacizumab-based maintenance therapy over discontinuation observation and found a superior effect of bevacizumab combined with 5-FU. The CAIRO 3 study (36) showed that bevacizumab plus capecitabine maintenance therapy significantly prolonged PFS compared with observation, but no significant difference was found in OS.

In summary, current evidence indicates a trend towards extending PFS through maintenance therapy, which patients also tolerate well. However, there is currently no compelling evidence supporting the effectiveness of maintenance therapy in prolonging OS, possibly due to various subsequent treatment regimens. The real-world study has shown that the two-stage maintenance strategy can benefit patients by consolidating efficacy while mitigating adverse reactions, with encouraging results for PFS and OS. However, clinicians should remain vigilant for adverse reactions in patients and be aware that cetuximab-based maintenance regimens may cause phenotypic conversion of the RAS gene. In addition, it is currently unclear which biological features are more likely to benefit from maintenance therapy. Several aspects, such as the optimal duration of induction therapy, the regimen and dosage of maintenance therapy, the duration of maintenance therapy, and the timing of reintroducing intensive chemotherapy, need further investigation.

## 5 Conclusion

In conclusion, cetuximab-based step-down maintenance therapy has shown potential benefits in terms of consolidating efficacy, reducing adverse reactions, and prolonging the survival of patients with unresectable mCRC who have received first-line cetuximab plus FOLFIRI and have RAS and BRAF wild-type tumors. Therefore, it is worthy of further exploration.

## Data availability statement

The original contributions presented in the study are included in the article/**Supplementary Material**. Further inquiries can be directed to the corresponding author.

## Ethics statement

The studies involving humans were approved by the Institutional Review Board (IRB number: 2020KY0144) of Fujian Medical University Union Hospital. The studies were conducted in accordance with the local legislation and institutional requirements. The participants provided their written informed consent to participate in this study. The study protocol was registered at the Chinese Clinical Trial Registry (Registration number: ChiCTR2000040940).

## Author contributions

TJ: Writing – original draft. HC: Conceptualization, Writing – original draft. XW: Data curation, Writing – original draft. FL: Formal analysis, Writing – original draft. HW: Investigation, Writing – original draft. JL: Methodology, Writing – original draft. XL: Writing – review & editing.

## Supplementary material

Supplementary Figure 1 Forest plot for the risk of disease progression in patients who received first-line chemotherapy with or without continuous anti-EGFR mAb therapy.

Supplementary Figure 2 Adverse reactions during Maintenance Phase 1.

Supplementary Figure 3 Adverse reactions during Maintenance Phase 2.

Supplementary Figure 4 The plot of overall patient survival. mOS: median overall survival.

Supplementary Table 1 Efficacy assessments by treatment period.

Supplementary Table 2 Cox regression multivariate analysis of the correlation between clinical data and PFS in patients.

Supplementary Table 3 Cox regression multivariate analysis of the correlation between clinical data and patient OS.
