# Supplementary figures and images for: A two-stage maintenance trial of cetuximab-based treatment in RAS and BRAF wild-type unresectable metastatic colorectal cancer: a retrospective real-world study
Source: Front Oncol. 2024 Jul 23;14:1425203. doi: 10.3389/fonc.2024.1425203 (PMC11300202; doi:10.3389/fonc.2024.1425203)

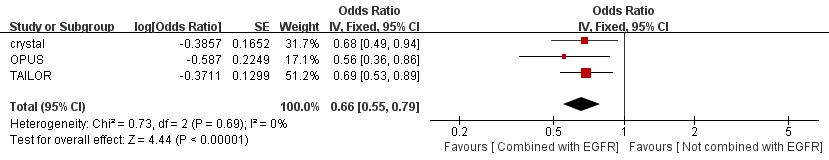

Supplement: Supplementary Figure 1 — Forest plot for the risk of disease progression in patients who received first-line chemotherapy with or without continuous anti-EGFR mAb therapy. [file Image_1.jpeg]

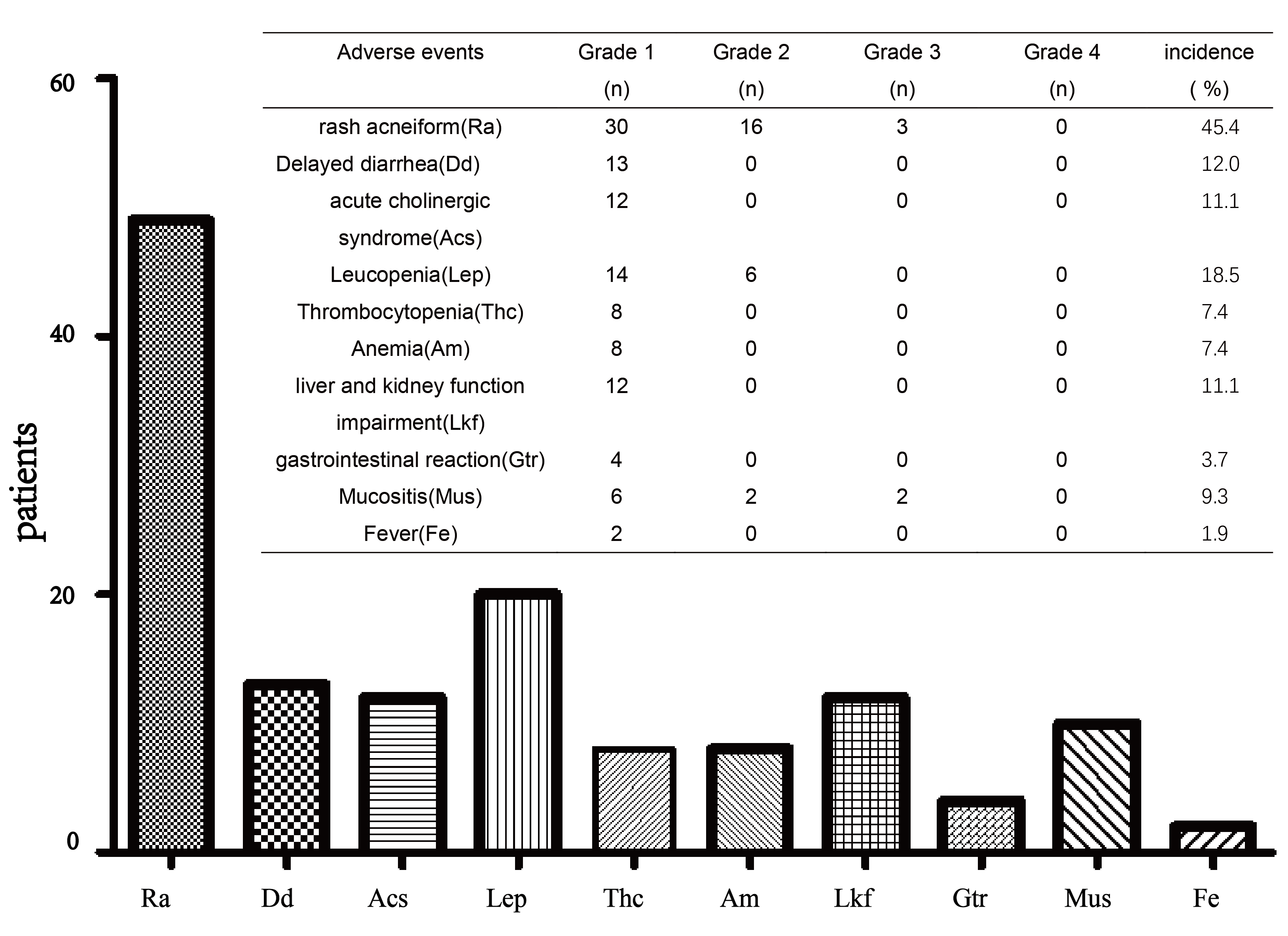

Supplement: Supplementary Figure 2 — Adverse reactions during Maintenance Phase 1. [file Image_2.jpeg]

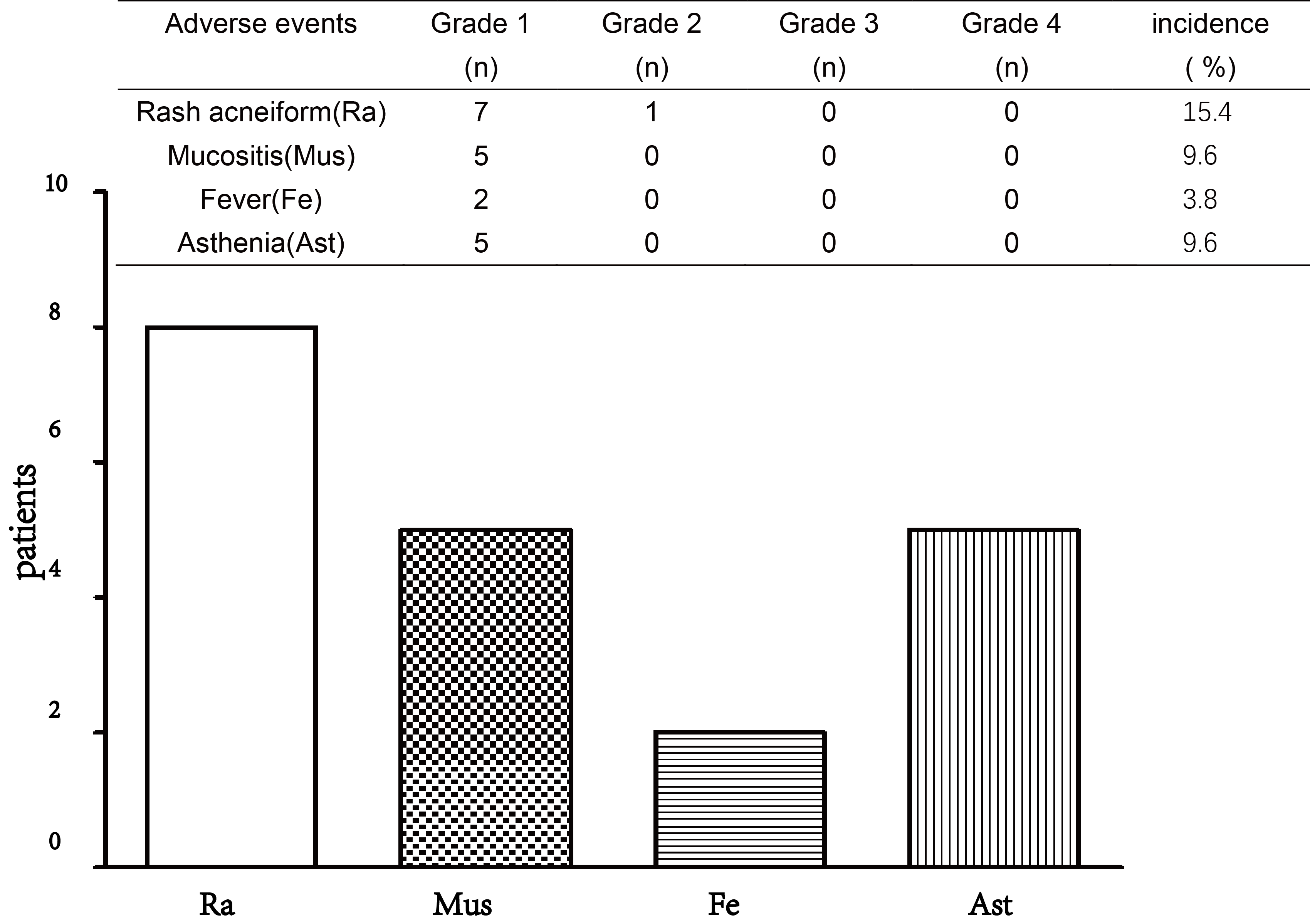

Supplement: Supplementary Figure 3 — Adverse reactions during Maintenance Phase 2. [file Image_3.jpeg]

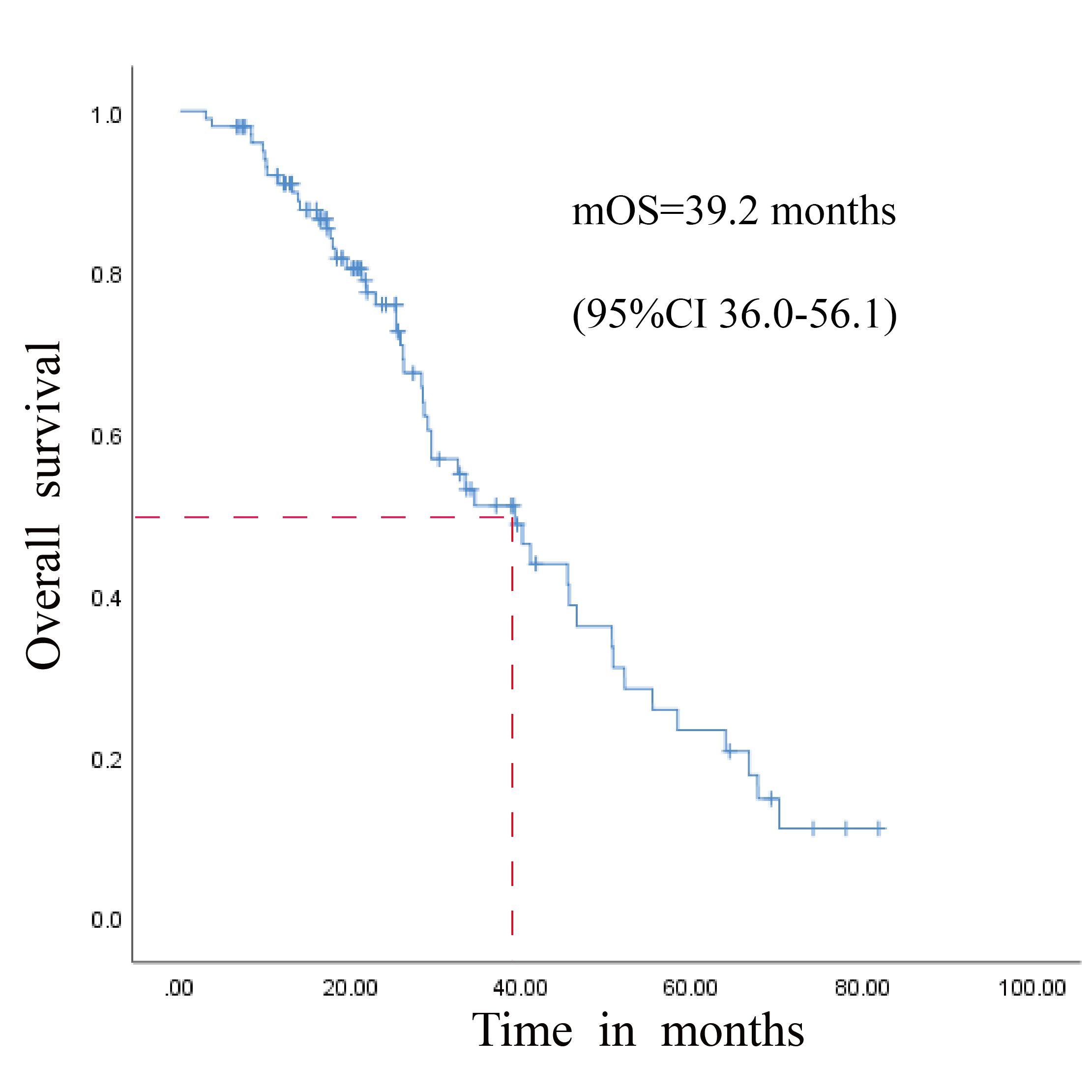

Supplement: Supplementary Figure 4 — The plot of overall patient survival. mOS: median overall survival. [file Image_4.jpeg]
