# Supplementary material for: A two-stage maintenance trial of cetuximab-based treatment in RAS and BRAF wild-type unresectable metastatic colorectal cancer: a retrospective real-world study
Source: Front Oncol. 2024 Jul 23;14:1425203. doi: 10.3389/fonc.2024.1425203 (PMC11300202; doi:10.3389/fonc.2024.1425203)
Supplement: Supplementary Table 1 — Efficacy assessments by treatment period. [file Table_1.docx]

|  | Maintenance 1  cetuximab+Irinotecan  (n=108) | Maintenance 2  cetuximab  (n=52) | Reinduction  cetuximab+FOLFIRI  (n=24) |
| --- | --- | --- | --- |
| DCR | 52(48.1%) | 17(32.7%) | 9(37.5%) |
| ORR | 12(11.1%) | 6 (11.5%) | 3(12.5%) |
