# Supplementary material for: A two-stage maintenance trial of cetuximab-based treatment in RAS and BRAF wild-type unresectable metastatic colorectal cancer: a retrospective real-world study
Source: Front Oncol. 2024 Jul 23;14:1425203. doi: 10.3389/fonc.2024.1425203 (PMC11300202; doi:10.3389/fonc.2024.1425203)
Supplement: Supplementary Table 2 — Cox regression multivariate analysis of the correlation between clinical data and PFS in patients. [file Table_2.docx]

| Group |  | No. subjects | PFS (95% CI) | HR(95%CI) | P value |
| --- | --- | --- | --- | --- | --- |
| Sex | Male | 66 | 12.070 (7.482-16.658) | 1.159 (0.694-1.935) | 0.572 |
|  | Female | 42 | 8.230 (2.565-13.895) |  |  |
| Age(years) | ＜60 | 66 | 10.230 (7.087-13.373) | 1.018(0.587-1.763) | 0.950 |
|  | ≥60 | 42 | 13.230 (8.281-18.179) |  |  |
| Primary tumor | Left colon | 96 | 11.570 (7.100-16.040) | 1.377(0.669-2.832) | 0.385 |
|  | Right colon | 12 | 8.100 (0.000-16.417) |  |  |
| Metastases | Lung | 13 | 12.070(4.262-19.878) | 0.990(0.442-2.220) | 0.981 |
|  | Liver | 47 | 15.170 (6.819-23.521) | 0.618(0.349-1.095) | 0.099 |
|  | Both lung and liver | 13 | 7.130 (2.625-11.635) | 1.382(0.610-3.128) | 0.438 |
|  | Other | 35 | 9.400 (7.089-11.711) |  |  |
| Primary tumor resection | Yes | 30 | 31.270(2.148-60.392) | 2.236(1.188-4.207) | 0.013 |
|  | No | 78 | 8.230 (6.570-9.890) |  |  |
| Local treatment of metastases | Yes | 42 | 20.630(11.925-29.335) | 0.553(0.319-0.959) | 0.035 |
|  | No | 66 | 7.700 (5.821-9.579) |  |  |
