# Supplementary material for: A two-stage maintenance trial of cetuximab-based treatment in RAS and BRAF wild-type unresectable metastatic colorectal cancer: a retrospective real-world study
Source: Front Oncol. 2024 Jul 23;14:1425203. doi: 10.3389/fonc.2024.1425203 (PMC11300202; doi:10.3389/fonc.2024.1425203)
Supplement: Supplementary Table 3 — Cox regression multivariate analysis of the correlation between clinical data and patient OS. [file Table_3.docx]

| Group |  | No. subjects | mOS (95% CI) | HR (95% CI) | P value |
| --- | --- | --- | --- | --- | --- |
| Sex | Male | 64 | 40.1(25.820-54.440) | 0.532 (0.269-1.052) | 0.070 |
|  | Female | 40 | 39.2(20.758-57.702) |  |  |
| Age(years) | ＜60 | 64 | 41.1(23.367-58.773) | 0.741(0.382-1.441) | 0.377 |
|  | ≥60 | 40 | 39.2(36.293-54.107) |  |  |
| Primary tumor | Left colon | 92 | 41.1(26.622-55.518) | 0.904(0.389-2.104) | 0.815 |
|  | Right colon | 12 | 25.3 (17.999-32.661) |  |  |
| Metastases | Lung | 12 | 29.5 (18.898-40.042) | 0.951(0.338-2.673) | 0.924 |
|  | Liver | 47 | 40.1 (10.812-69.448) | 0.531(0.284-1.003) | 0.051 |
|  | Both lung and liver | 11 | NR | 0.768(0.247-2.388) | 0.648 |
|  | Other | 34 | 39.2(29.605-48.855) |  |  |
| Primary excision | Yes | 29 | 50.6(40.574-60.626) | 0.308(0.138-0.690) | 0.004 |
|  | No | 75 | 29.5(24.823-34.117) |  |  |
| Local treatment of metastases | Yes | 39 | 46.5(31.135-61.805) | 1.519(0.777-2.970) | 0.222 |
|  | No | 65 | 29.5(23.269-35.671) |  |  |
